# Neuropsychological and Emotional Correlates of Personality Traits in Parkinson’s Disease

**DOI:** 10.3233/BEN-129017

**Published:** 2013

**Authors:** Janneke Koerts, Lara Tucha, Klaus L. Leenders, Oliver Tucha

**Affiliations:** ^1^Department of Clinical and Developmental NeuropsychologyUniversity of GroningenGroningenThe Netherlands; ^2^Department of Neurology, University Medical Center GroningenUniversity of GroningenGroningenThe Netherlands

**Keywords:** Novelty seeking, harm avoidance, executive functions, depression, Parkinson’s disease

## Abstract

Parkinson’s disease (PD) is, apart from the well-known motor symptoms, also characterized by neuropsychological and emotional disturbances. However, patients also often present with a personality profile of low Novelty Seeking and high Harm Avoidance. This profile can be identified as the disease emerges, which raises the question whether these traits correlate with more fundamental neuropsychological and emotional disturbances. This study determined the neuropsychological and emotional correlates of Novelty Seeking, Harm Avoidance and two other personality traits that are often considered in PD, i.e. Reward Dependence and Persistence.

Forty-three patients and 25 healthy participants were assessed with the Temperament and Character Inventory, a symptoms of depression questionnaire and neuropsychological tests.

PD patients showed a higher Harm Avoidance than healthy participants, which was predicted by symptoms of depression. Groups did not differ regarding Novelty Seeking, Reward Dependence and Persistence. While cognitive flexibility was a predictor of Reward Dependence, Persistence was predicted by divergent thinking and inhibition. Novelty Seeking was not predicted by cognition or emotion.

In conclusion, cognition and emotion are selectively related to personality traits in PD. Whereas Harm Avoidance covaries with emotional symptoms, Persistence and Reward Dependence are related to cognition. Alterations in personality, cognition and emotion in PD are thus not independent from each other.

## 1. Introduction

Parkinson's disease (PD) is a progressive neurodegenerative disease that is characterized by motor symptoms and non-motor symptoms, such as depression and cognitive impairments in the domains of executive functions, memory and psychomotor speed [1–5]. Since the first half of the 20th century, however, it was repeatedly suggested that PD is also associated with a typical (premorbid) personality profile of inflexibility, cautiousness, industriousness and low impulsivity [6]. Recently, Poletti and Bonuccelli [6] published an overview of the research examining personality traits in patients with PD and concluded that, in contrast to what was previously expected, there is no robust support for a characteristic premorbid personality profile in patients with PD. This lack of support is caused by the fact that it is difficult to assess premorbid personality traits with current test instruments. Furthermore, studies used different instruments for the measurements of personality traits and revealed only conflicting results. Poletti and Bonuccelli did however report that once PD emerges patients show two typical personality traits [6]. First, PD patients have often been described as "low Novelty Seekers", i.e. they prefer to stay with familiar routines, are orderly, disciplined, reserved and controlled, have a preference for saving and spending according to bud-

ISSN 0953-4180/13/$27.50 © 2013 – IOS Press and the authors. All rights reserved gets and require considerable time when making decisions [7]. Second, PD patients have been characterized by high levels of Harm Avoidance. According to this trait, PD patients have been described as inhibited, tense, pessimistic, easily tired and slow to recuperate from physical exertion [7]. Further personality traits that have been studied in PD are Reward Dependence (i.e. the tendency to respond to signals of reward (e.g. social approval) and the maintenance of behavior that was previously associated with reward or relief of punishment) [7] and Persistence (i.e. the ability to continue, expect and seek reward, even when the expected outcome is not always or only rarely successful) [8]. However, no specific profiles could be detected in PD patients with regard to these traits [6].

Since no premorbid personality profile was found in patients with PD, it appears that patients change in personality (i.e. low Novelty Seeking and high Harm Avoidance) as the disease emerges or evolves. This, however, raises the question whether these personality traits can be understood as changes associated with more fundamental neuropsychological impairments and emotional disturbances, which are already present in newly diagnosed patients with PD [4]. Studies reported cognitive impairments in about 30% of newly diagnosed patients [4] which primarily affect executive functions and psychomotor speed. Executive functioning is an umbrella term encompassing different functions which are needed in non-routine situations including cognitive flexibility, inhibition and planning [9]. Based on the descriptions of Novelty Seeking and Harm Avoidance as mentioned above, one could expect that Novelty Seeking is related to executive functions and psychomotor speed, whereas Harm Avoidance is associated with symptoms of depression and performance of tests of executive functions (i.e. inhibition). The aim of this study was to investigate the neuropsychological and emotional correlates of Novelty Seeking, Harm Avoidance, Reward Dependence and Persistence in PD.

## 2. Methods

### 2.1. Participants

Patients were randomly selected from the PD patients visiting the Movement Disorders outpatient clinic of the Department of Neurology of the University Medical Center Groningen (UMCG), The Netherlands. All selected patients were contacted by the researcher (JK) and provided with information about this study. Forty-three out of 74 PD patients agreed upon participation and all signed an informed consent prior to study inclusion. Reasons for rejection of participation included time constraints ($n = 11$), disinterest in supporting research ($n = 12$) and a poor health condition ($n = 8$).

Table 1
Descriptive and disease characteristics of PD patients ($n = 43$) and healthy participants ($n = 25$)

| | PD patients M (SD) | Healthy participants M (SD) |
|---|---|---|
| Age (years) | 63.6 (8.8) | 62.8 (11.5) |
| Education* | 5.3 (1.1) | 4.8 (0.7) |
| Gender (female/male) | 19/24 | 14/11 |
| MMSE total | 27.5 (1.5) | 27.6 (1.2) |
| Disease duration (years) | 5.3 (4.2) | |
| UPDRS, part III | 24.6 (8.8) | |
| H&Y | 2.2 (0.6) | |
| LEDD | 583.7 (435.7) | |

*Dutch education scale ranging from 1 (elementary school not finished) to 7 (university degree); MADRS = Montgomery-Åsberg Depression Rating Scale; MMSE = Mini Mental State Examination; UPDRS = Unified Parkinson's Disease Rating Scale; H&Y = Hoehn and Yahr scale; LEDD = Levodopa Equivalent Daily Dose.

Patients were diagnosed with idiopathic PD according to the criteria of the UK Parkinson's Disease Society Brain Bank. The motor severity of symptoms was assessed with the Unified Parkinson's Disease Rating Scale [10] and the Hoehn and Yahr scale (H&Y) [11]. A Levodopa Equivalent Daily Dose (LEDD) was calculated for all patients [12]. Patients were assessed in their regular on-state following medication.

Healthy participants were recruited from the Groningen community through word-of-mouth or were related to other participants. The same procedure was followed as in patients. Twenty-five healthy participants agreed upon participation and signed an informed consent prior to study inclusion. Level of education was rated for all participants with a Dutch education scale, ranging from 1 (elementary school not finished) to 7 (university degree). Groups did not differ in age ($t = 0.30$; $p = 0.76$), gender (Chi-Square = 0.88; $p = 0.35$) and education level ($Z = -1.58$; $p = 0.11$). Descriptive and disease characteristics of PD patients and healthy participants are reported in Table 1. Exclusion criteria were dementia (MMSE < 24) [13], neurological disorders other than idiopathic PD, co-morbidities according to medical records of patients, deep brain stimulation and the use of drugs (other than anti-parkinsonian drugs) known to influence cognition. PD patients with a clinical depression according to the criteria of Leentjens et al. [14] were not excluded from the present study. This study was approved by the medical ethical committee of the UMCG.

## 2.2. Measures

### 2.2.1. Personality questionnaire

Personality characteristics of participants were assessed with a shortened version of the Temperament and Character Inventory (TCI-125) [15]. The TCI-125 is a true/false questionnaire which is based on the Psychobiological Model of Temperament and Character of Cloninger et al. [8]. Seven personality traits are measured, including Novelty Seeking, Harm Avoidance, Reward Dependence, Persistence, Self-Directiveness, Cooperativeness and Self-Transcendence. The present study focuses only on Novelty Seeking, Harm Avoidance, Reward Dependence and Persistence which have been shown to be the most relevant personality traits with regard to PD.

### 2.2.2. Symptoms of depression questionnaire

Symptoms of depression were assessed with the Montgomery-Åsberg Depression Rating Scale (MADRS) [16]. This scale contains items that measure affective symptoms (e.g. reported sadness and pessimistic thoughts) as well as items measuring somatic symptoms (e.g. reduced sleep and lassitude) and cognitive symptoms (e.g. concentration difficulties) of depression.

### 2.2.3. Tests of executive functions

Several standardized, reliable and valid neuropsychological test measures of executive functions were applied in this study [9].

The Stroop Color Word Test [17] is used to assess the ability of a person to maintain a goal in mind and to suppress a habitual response in favor of a less familiar one (i.e. inhibition). This test contains three cards, the Word card, the Color card and the Color-Word card. The target measure for inhibition is the Color-Word card. This task requires participants to suppress the automatic tendency to read, while naming the color of words that are themselves color names. The performance was corrected for psychomotor speed, by dividing the time needed for the Stroop Color-Word card by the time needed for the Stroop Color card.

Cognitive flexibility was assessed with the Trail Making Test [18] which consists of two parts. Part A requires participants to draw a line, as fast as possible, between numbers in ascending order. In part B, numbers and letters are used and participants need to switch attention between both concepts: they have to draw a line between both types of stimuli in ascending order, alternating between numbers and letters and also as fast as possible. The target measure for cognitive flexibility was the performance on part B.

The Odd Man Out [19] was also used to measure cognitive flexibility. This test requires participants to indicate which shape, out of a set of four shapes, is different. Three selection rules are possible and two sets of twelve cards are used. For each set of cards participants have to specify a different rule and both sets are alternated four times. The total number of incorrect responses is calculated.

The Digit Span Backward of the Wechsler Memory Scale–Revised [20] was used to assess verbal working memory. A series of digits are read to the participants who are required to repeat the digits in the reverse order.

Semantic and phonemic verbal fluency tests were used to evaluate divergent thinking. The semantic verbal fluency test requires participants to produce as many animals or professions as possible, each within a time interval of one minute. Participants are not allowed to name the same word twice. The total number of correctly produced animals and professions is registered. During the phonemic verbal fluency test, participants are asked to produce as many words as possible within one minute starting with the letters D, A or T [21,22]. This verbal fluency test is equivalent to the FAS-test as devised by Benton and deHamser [23]. Participants are not allowed to name the same word twice or to produce names of persons or towns. The total number of correctly produced words starting with D, A and T were calculated and combined into one phonemic verbal fluency score.

The Zoo-Map of the Behavioral Assessment of the Dysexecutive Syndrome [24] was used to assess planning. Participants were required to plan a route through a zoo visiting previously specified attractions and following a set of rules. The test consists of two parts. The first part is presented as an unstructured situation, participants are not provided with information that could help planning a route and only the attractions that need to be visited and the rules are presented. In contrast, part two is structured. An action plan which needs to be followed is provided in addition to the same set of rules as in part one. Both parts are rated by calculating the total number of correctly visited attractions minus errors (e.g. visiting attractions that were not previously specified, not obeying the rules).

Finally the Frontal Assessment Battery which assesses different executive functions, including conceptualization, cognitive flexibility, motor programming, sensitivity to interference and inhibitory control was used [25]. Each subtest is scored between 0 and 3. A total score is calculated by adding the scores on the different subtests.

Table 2
Scores of PD patients ($n = 43$) and healthy participants ($n = 25$) on Novelty Seeking, Harm Avoidance, symptoms of depression questionnaire as well as tests of executive functions and psychomotor speed

| | PD patients M (SD) | Healthy participants M (SD) | t | p | d |
|---|---|---|---|---|---|
| *Personality* | | | | | |
| Novelty Seeking | 8.2 (2.5) | 7.4 (2.3) | 1.24 | 0.22 | 0.33 |
| Harm Avoidance | 10.7 (4.2) | 8.4 (4.2) | 2.13 | 0.04* | 0.55 |
| Reward Dependence | 8.6 (2.7) | 9.2 (2.4) | −0.90 | 0.37 | 0.23 |
| Persistence | 2.5 (1.4) | 2.2 (1.6) | 0.71 | 0.48 | 0.20 |
| *Executive functions* | | | | | |
| FAB | 15.2 (2.5) | 17.4 (0.9) | −5.17 | < 0.001*** | 1.06 |
| Stroop interference index | 1.7 (0.3) | 1.6 (0.2) | 1.42 | 0.16 | 0.37 |
| Trail Making Test B | 127.2 (58.1) | 102.6 (68.4) | 1.58 | 0.12 | 0.40 |
| OMO | 5.2 (5.6) | 1.2 (1.4) | 4.42 | < 0.001*** | 0.88 |
| Digit Span backward | 5.6 (2.1) | 6.1 (1.4) | −1.01 | 0.32 | 0.27 |
| BADS Zoo map | 2.4 (1.2) | 2.8 (0.9) | −1.49 | 0.14 | 0.36 |
| Semantic fluency | | | | | |
| –Animals | 20.9 (5.4) | 21.6 (4.4) | −0.58 | 0.57 | 0.14 |
| –Professions | 15.0 (4.4) | 17.5 (3.8) | −2.42 | 0.02* | 0.60 |
| Letter fluency | 38.7 (14.9) | 38.6 (10.5) | 0.01 | 0.99 | 0.00 |
| *Psychomotor speed* | | | | | |
| Trail Making Test A | 52.3 (19.1) | 42.1 (28.0) | 1.78 | 0.08 | 0.45 |
| Stroop Word Card | 51.3 (12.5) | 50.8 (11.4) | 0.17 | 0.86 | 0.04 |
| *Depression* | | | | | |
| MADRS | 9.2 (7.1) | 2.3 (2.1) | 4.80 | < 0.001*** | 1.19 |

* $\leqslant$ 0.05; ** $\leqslant$ 0.01; *** $\leqslant$ 0.005. BADS = Behavioral Assessment of the Dysexecutive Syndrome; FAB = Frontal Assessment Battery; MADRS = Montgomery-Åsberg Depression Rating Scale; OMO = Odd Man Out.

#### 2.2.4. *Tests of psychomotor speed*

The time needed for completion of Part A of the Trail Making Test [18] and of the Stroop Word Card [17] were used as a measure of psychomotor speed.

### 2.3. *Statistical analyses*

Predictive Analytic SoftWare (PASW) 18.0 was used for statistical analyses. To compare PD patients and healthy control participants with regard to Novelty Seeking, Harm Avoidance, Reward Dependence, Persistence, as well as executive functions, psychomotor speed and symptoms of depression, t-tests for independent samples were used. Furthermore, patients' performances in tests of executive functioning and psychomotor speed were compared to normative data as provided by the authors of tests applied or the authors of published normative datasets as used in clinical practice [9]. In accordance with the new diagnostic criteria for mild cognitive impairment in PD of the Movement Disorders Society Task Force a cognitive test performance was classified as "being impaired" when the standard score was 1–2 standard deviations below the mean [26]. Subsequently, it was calculated on how many tests of executive functions as well as on how many tests of psychomotor speed patients showed impairments. With regard to the symptoms of depression, patients were classified as clinically depressed or non-depressed according to the criteria of Leentjens et al. [14].

Multiple linear regression analyses (method: stepwise) were performed in the group of PD patients to determine the unique contribution of symptoms of depression, executive functions, psychomotor speed and disease characteristics of PD (i.e. disease duration, H&Y score, UPDRS part III score and LEDD) to Novelty Seeking, Harm Avoidance, Reward Dependence or Persistence. A p-value of $\leqslant 0.05$ was considered significant.

## 3. Results

PD patients showed significantly higher scores on Harm Avoidance than healthy control participants. No differences were found between these groups with regard to Novelty Seeking, Reward Dependence and Persistence (Table 2). With regard to cognition, PD patients performed significantly worse than healthy participants on measures of executive functioning including the Frontal Assessment Battery, a semantic fluency test (i.e. professions) and the Odd Man Out. Furthermore, PD patients reported more symptoms of depression than healthy control participants and 19% of patients ($n = 8$) were classified as clinically depressed.

The comparison of patients' test performance with normative data revealed that 74% of patients showed impairments in executive functioning. While 21% of patients ($n = 9$) showed an impairment in one test measuring executive functions, 21% ($n = 9$) displayed impairments on two test measures, 9% ($n = 4$) on three, 5% ($n = 2$) on four, 16% ($n = 7$) on five and 2% ($n = 1$) on all test measures applied. Regarding psychomotor speed, the results showed that 54% of patients showed impairments on one or two tests of psychomotor speed. The remaining 46% of patients showed no impairments in this domain.

The results of the multiple regression analyses showed that performances on tests of executive functions and psychomotor speed as well as symptoms of depression and disease characteristics did not predict Novelty Seeking in PD patients. Harm Avoidance was, however, significantly predicted by the symptoms of depression (i.e. score on the MADRS; $R^2 = 0.20$, $F = 8.97$, $p = 0.005$). Furthermore, while the performance on part B of the Trail Making Test represented a significant predictor of Reward Dependence ($R^2 = 0.13$, $F = 5.48$, $p = 0.03$), Persistence was significantly predicted by semantic fluency performance (i.e. professions) and the Stroop interference index ($R^2 = 0.27$, $F = 6.46$, $p = 0.004$), explaining 15% and 12% of variance respectively.

## 4. Discussion

The results of the present study suggest that alterations of cognition, emotion and personality in patients with PD are not independent from each other. This is in accordance with previous findings demonstrating that cognition and emotion sub-serve personality traits in patients with other neurodegenerative disorders, including fronto-temporal dementia, Alzheimer's disease, dementia with Lewy bodies and progressive supranuclear palsy [27]. However, the results of the present study also indicate that there are selective effects of emotion and cognition on personality in PD. While some personality traits are associated with cognition but not emotion, others have been shown to be related with emotion but not cognition.

With regard to emotion, an association was found between symptoms of depression in PD and Harm Avoidance but not with Novelty Seeking, Reward Dependence and Persistence. The present sample of PD patients scored significantly higher on Harm Avoidance and reported significantly more symptoms of depression than healthy control participants, with a clinical but mild depression being present in 19% of patients. A relation between PD and affective disorders has often been reported [28–30] with prevalence rates of depression ranging between 30 and 35% [31]. Higher levels of Harm Avoidance suggest that PD patients are more inhibited and tense (even in normal circumstances), worry frequently, are pessimistic, negative and recuperate slower from physical exertion and emotional stress than healthy individuals [7]. Indeed, other studies focusing on PD [32,33] and studies performed on the general population [34–36] also revealed that Harm Avoidance has a strong association with depression and anxiety. The nature of the relation between Harm Avoidance and affective disorders in PD is however not clear. One could consider that Harm Avoidance represents a personality trait of PD patients. However, since affective disorders do not necessarily follow the progressive neurodegenerative course of PD and because affective disorders can sometimes be successfully treated in PD [38], one can also argue that Harm Avoidance is not a personality trait but state dependent instead [6]. The relation between Harm Avoidance and depression in PD thus needs further exploration in future studies.

Regarding cognition, significant associations were found with Persistence and Reward Dependence, but not with Harm Avoidance and Novelty Seeking. The present sample of PD patients was characterized by frequent cognitive impairments and significant deficits in the domain of executive functions (i.e. Frontal Assessment Battery), including divergent thinking (i.e. fluency professions) and cognitive flexibility (i.e. Odd Man Out). However, even though Persistence and Reward Dependence are both personality traits that are associated with cognitive functioning in PD, the present as well as previous studies [40–43] indicate that these traits are not specific for PD, because PD patients did not differ from healthy participants in these traits. Nevertheless, lower levels of Persistence in PD were associated with difficulties in divergent thinking and the ability to inhibit behavior. This might indicate a complex interaction between cognition and Persistence, which might be explained by the attributes ascribed to low Persistence. Cloninger [8] described individuals with low Persistence as being changeable, irresolute and easily discouraged as well as having difficulties with goal-oriented behavior. While the latter three characteristics are considered to be detrimental for performance, changeability can also be a beneficial feature. There are many situations in which a change of behavior would be more beneficial and adaptive to situational demands, e.g. in situations with rapidly changing reward contingencies [44]. Therefore it is not surprising that both a better ability to inhibit behavior (i.e. enabling changeability) as well as difficulties in divergent thinking (i.e. hampering goal-directedness and resoluteness) are associated with low Persistence.

Reward Dependence was associated with cognitive flexibility (i.e. Trail Making Test part B), with difficulties in cognitive flexibility being related to higher levels of Reward Dependence. This association appears reasonable considering that flexibility in general is a crucial component of independence. People with high levels of Reward Dependence who are sensitive to emotional cues, responsive to social pressure as well as crave for gratification may therefore be less independent and depend more on the social support of others [7]. The relation between Reward Dependence and cognitive flexibility might also be explained in terms of the brain structures involved in their processing. Research on healthy individuals [45] as well as PD patients [46] revealed that both the striatum and the prefrontal cortex are involved in cognitive flexibility. Indeed, recent imaging studies demonstrated that these structures are relevant for Reward Dependence with Reward Dependence being associated with connectivity in the white matter of the frontal lobe and with the strength of the relative connectivity between the striatum and prefrontal regions [47,48].

A final personality trait investigated in the present study is Novelty Seeking. A low Novelty Seeking was previously described as a characterizing personality trait of PD patients [6]. Nevertheless, the present study, just like other studies [40,41], did not find a difference between patients with PD and healthy participants in Novelty Seeking. This might be explained by the composition of the present sample. Tomer and Aharon-Peretz [42] revealed that only patients with right onset of the disease (i.e. motor symptoms initially presented on the right side of the body) showed a low Novelty Seeking, whereas only patients with a left onset of the disease showed a high Harm Avoidance. Considering that the present study found a high Harm Avoidance, it might be possible that the majority of PD patients included in this study had a left onset of the disease. This, however, remains speculative since the side of disease onset was not assessed. Another speculation for not finding differences between groups regarding Novelty Seeking might refer to the presence of impulsive compulsive behaviors in PD patients. Impulsive compulsive behaviors have been shown to be strongly associated with high Novelty Seeking. Furthermore, these behaviors have been found in about 14% of patients with PD treated with dopamine agonists [49,50]. Despite us trying to exclude patients with co-morbidities including impulsive compulsive behaviors, the possibility exist that patients did not report these behaviors to the consultant neurologist, in particular because these types of problems are often hidden or unnoticed by patients [51]. Novelty Seeking was not found to be related with cognitive impairments and emotional disturbances of PD patients. It is difficult to discuss this finding, since a non-significant result does not mean that there is no association. One could assume that the selection of measures as applied in the present study did lack a sensitive measure. In particular, a measure assessing reward and punishment processing appears relevant since a study on the effects of dopamine agonists reported associations between Novelty Seeking and reward learning as well as punishment processing [43]. Therefore, future studies on personality in PD should consider including measures on reward and punishment processing.

A limitation of the present study was that PD patients were compared to healthy participants and not to patients with other progressive degenerative disorders. It is therefore not clear to what extent the results are specific to PD.

In conclusion, the results of this study suggest that cognition and emotion are selectively related to personality traits in PD. Whereas Harm Avoidance covaries with the emotional symptoms that can accompany PD, Persistence and Reward dependence are related to cognition in PD. Alterations in personality, cognition and emotion in PD are thus not independent from each other.
